# Global obesity epidemic and rising incidence of early-onset cancers

**DOI:** 10.7189/jogh.14.04205

**Published:** 2024-10-11

**Authors:** Jianjiu Chen, Piero Dalerba, Mary Beth Terry, Wan Yang

**Affiliations:** 1 Department of Epidemiology, Mailman School of Public Health, Columbia University, New York, New York, USA; 2 Herbert Irving Comprehensive Cancer Center (HICCC), Columbia University Irving Medical Center, New York, New York, USA; 3 Department of Pathology and Cell Biology, Columbia University, New York, New York, USA; 4 Division of Digestive and Liver Disorders, Department of Medicine, Columbia University Irving Medical Center, New York, New York, USA; 5 Digestive and Liver Disease Research Center (DLDRC), Columbia University Irving Medical Center, New York, New York, USA; 6 Columbia Stem Cell Initiative (CSCI), Columbia University Irving Medical Center, New York, New York, USA; 7 Center for Discovery and Innovation (CDI), Hackensack Meridian Health, Nutley, New Jersey, USA

## Abstract

**Background:**

Incidence of early-onset cancers at multiple organ sites has increased worldwide in recent decades. We investigated whether such increasing trends could be explained by trends in obesity.

**Methods:**

We obtained incidence data for 21 common cancers among 25–49-year-olds during 2000–2012 in 42 countries from the Cancer Incidence in Five Continents database. Nine cancers we examined have been classified as obesity-related by the International Agency for Research on Cancer. Estimates of overweight and obesity prevalence came from the Non-communicable Disease Risk Factor Collaboration. Using country-level data, we examined whether changes in the prevalence of overweight and obesity combined were correlated with changes in cancer incidence, after accounting for various time lags (0–15 years) between exposure and cancer diagnosis. To test the validity of our approach, we conducted negative control analyses (using non-obesity-related cancers as the outcome variable, and per-capita gross national income as the exposure variable), and sensitivity and supplemental analyses using alternative data streams or processing.

**Results:**

We found increased incidence for six of nine obesity-related and seven of twelve non-obesity-related cancers in 25–49-year-olds. These increases were more predominant in Western countries (particularly Australia, the USA, Canada, Norway, the Netherlands, and Lithuania). For four obesity-related cancers displaying increased incidence (colon, rectum, pancreas, kidney), changes in cancer incidence were positively correlated with changes in overweight and obesity prevalence. When accounting for a 15-year lag, the estimated correlation was 0.27 (95% confidence interval (CI) = −0.04, 0.53; *P* = 0.090) for colon cancer, 0.33 (95% CI = 0.02, 0.58; *P* = 0.036) for rectal cancer, 0.39 (95% CI = 0.08, 0.64; *P* = 0.018) for pancreatic cancer, and 0.22 (95% CI = −0.10, 0.50; *P* = 0.173) for kidney cancer. Similar correlations were found in the sensitivity and supplemental analyses. We did not find similar correlations with excess body weight for the non-obesity-related early-onset cancers, nor correlations with per-capita gross national income for any cancer types, in the negative control analyses.

**Conclusions:**

Worldwide increases in early-onset colon, rectal, pancreatic, and kidney cancers may have been partly driven by increases in excess body weight. The increases in other early-onset cancers, however, were likely driven by other factors deserving of further investigation.

Growing evidence has shown that early-onset cancers – typically defined as cancers diagnosed before age 50 – have increased in incidence worldwide in recent decades across multiple organ sites [1–8]. Because many of these cancers (e.g. colorectal, pancreatic, and kidney cancers) have been associated with obesity [1,9,10] and because the prevalence of obesity has increased globally in past decades [11,12], several studies have hypothesised that the recent increase in the incidence of early-onset cancers might be a direct consequence of the obesity epidemic [9,13,14]. For the causal links between obesity and cancer risk, there are several proposed mechanisms involving complex interactions [15,16]. In essence, excess body weight and the resulting excess adipose tissue could alter biochemistry (particularly, hormones and cell-signalling molecules) that could affect cancer risk; this includes causing insulin resistance, elevated level of leptin (a hormone that regulates food intake but could also stimulate cell proliferation and tumour growth), adipokines-induced inflammation, and increased fatty acid metabolism, all of which could increase cancer risk [15,16]. The strength of the association between obesity and increased cancer incidence, however, remains somewhat uncertain. This is because a large number of epidemiological and societal factors have changed over the last few decades, often with trends that are parallel in time to those of obesity and cancer incidence. Furthermore, although many epidemiological studies have identified excess body weight as a risk factor for cancers occurring later in life (mostly above age 50) [10], much fewer studies have focused on early-onset cancers [17]. Even fewer studies have examined the impact of excess body weight during early life (e.g. adolescence) [18-20] – a potentially critical period for early-onset cancer development [13,17].

This study investigated the impact of the obesity epidemic on early-onset cancers by leveraging data for body weight and cancer incidence in the 42 countries in the Cancer Incidence in Five Continents (CI5plus) database. We reasoned that, for a given cancer with increased incidence among young adults, if the obesity epidemic was a key driver, populations having larger increases in body weight would tend to have larger increases in cancer incidence. Therefore, we would expect the magnitude of country-level changes in obesity prevalence to be correlated (with a lag) with the magnitude of changes in cancer incidence, resembling a dose-response relationship (**Figure 1**, Panel A). However, if the increased cancer incidence was not driven by but simply coincided with the obesity epidemic, we would expect such country-level correlations to be minimal (**Figure 1**, Panel B). Following this rationale, our analysis included 21 common cancers in young adults, regardless of their association with obesity found in older adults; we examined whether changes in the incidence of these cancers were correlated with changes in the prevalence of overweight and obesity (since adolescence) among the 42 countries. In addition, we conducted a comprehensive set of negative control, sensitivity, and supplemental studies to test the validity of our approach and findings.

**Figure 1:**
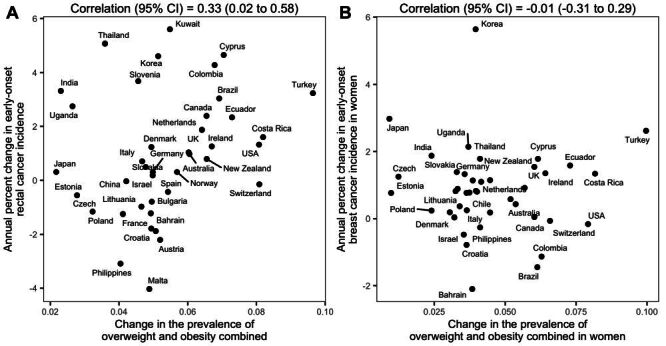
Illustration of study design using rectal cancer and female breast cancer as examples. To limit spurious correlation, we examine whether the magnitude of country-level changes in cancer incidence scaled with the magnitude of changes in body weight in a dose-response fashion (i.e. indicative of an association). For rectal cancer (classified as obesity-related), country-level changes in early-onset cases (y-axis) were positively correlated with changes in body weight (**Panel A**). In contrast, for pre-menopausal female breast cancer (not classified as obesity-related), there was no positive correlation (**Panel B**). Both analyses accounted for a 15-year lag between the exposure (i.e. changes in body weight for 20–34-year-olds during 1985–1997) and the outcome (i.e. changes in cancer incidence for 35–49-year-olds during 2000–2012).

## METHODS

### Data

Cancer incidence data came from the CI5plus database, which was the largest global cancer database providing comparable cancer incidence data across populations and time [21]. The CI5plus database included data from cancer registries in 42 countries (Figure S1 in the **Online Supplementary Document**), and in 20 countries, the registries covered the entire population [22]. For countries without such a complete coverage (Table S1 in the **Online Supplementary Document**), we combined data from all available registries for that country. The CI5plus annual cancer incidence data dated back to 1998 for all participating countries (and earlier for a subset) and were available through 2012 (Table S1 in the **Online Supplementary Document**). Cancer incidence rates were age-standardised to the 1966 Segi-Doll world standard population [23].

We included 21 cancer types (based on the International Classification of Disease, 10th revision (ICD-10)), for which the median incidence rate across countries exceeded one per 100 000 person-years among 25–49-year-olds during 2000–2012. The included cancers covered 89% of all reported cases among the study population. Nine of these cancer types have been classified as obesity-related based on a review conducted by the International Agency for Research on Cancer [10]. The review included both epidemiological evidence, which documented the association between obesity and cancers in cohort and case-control studies, and experimental evidence, which provided mechanistic insights into these associations [10]. The nine obesity-related cancers were cancers of the colon (ICD-10 code: C18), rectum (C19-20), liver (C22), pancreas (C25), corpus uteri (C54), ovary (C56), kidney (C64-65), thyroid (C73), and stomach (C16). The remaining, non-obesity-related types (included as negative controls) were cancers of the oral cavity and pharynx (C00-14), lung (C33-34), premenopausal breast (C50), cervix uteri (C53), prostate (C61), testis (C62), bladder (C67), brain and other central nervous system (C71-72), melanoma of the skin (C43), Hodgkin lymphoma (C81), non-Hodgkin lymphoma (C82-86, C96), and leukaemia (C91-95). For corpus uterine cancers, we restricted the analysis to 29 countries, where the number of unspecified cases (i.e. cases at either cervix or corpus uteri but unknown) was minimal


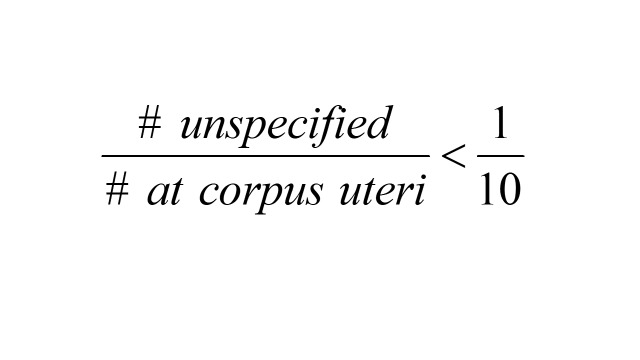
.

For cervix uterine cancer, we likewise restricted the analysis to 40 countries.

We obtained estimates of overweight and obesity prevalence from the Non-communicable Disease Risk Factor Collaboration (NCD-RisC) [11] and the Global Burden of Disease (GBD) study, separately [24]. These estimates were available for each country, sex, age, and year during 1980–2015. For adults aged 20 or above, both studies defined overweight as body mass index (BMI)≥25 and <30, and obesity as BMI≥30. For those aged under 20, the NCD-RisC defined overweight as BMI being 1 to 2 standard deviation (SD) above the median of the World Health Organization growth reference, and defined obesity as more than 2 SD above the median; the GBD study defined overweight and obesity using BMI cut-off points proposed by the International Obesity Task Force [25]. All prevalence estimates were age-standardised to the 1966 world standard population, as done for cancer incidence rates.

### Statistical analysis

Given the increasing trends of both body weight and many cancers, there would likely be spurious correlations between time series of overweight and obesity prevalence and cancer incidence in a given country (Figure S2 in the **Online Supplementary Document**). To limit such spuriousness, and in view of the substantial between-country heterogeneities in changes in body weight and cancer incidence, here we examined whether changes in body weight were correlated with changes in cancer incidence across the 42 countries.

As done in previous ecologic studies [26–29], we modelled the relationship between the exposure (here overweight and obesity prevalence; X) and the outcome (here cancer incidence rate; Y) using a generalised linear model, log(*Y*) ~ *X*. This model implies



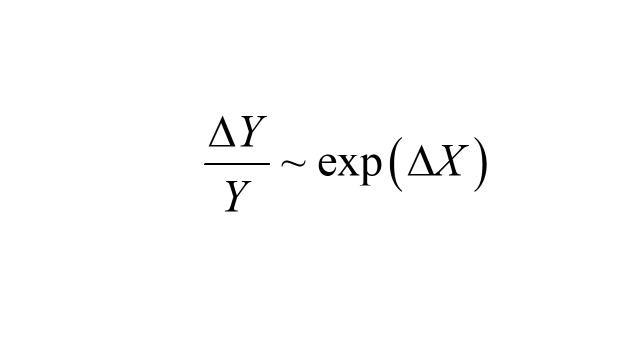



meaning that the relative change in cancer incidence rate(∆*Y*/*Y*) would vary log-linearly with the change in overweight and obesity prevalence (∆*X*). Thus, to examine the relationship between excess body weight and early-onset cancers, for each cancer, we first computed the average annual percent change in incidence rate (i.e. ∆*Y/Y*; using joinpoint analysis [30,31]) and the change in overweight and obesity prevalence (i.e. ∆*X*) for each country, and then pooled these measures for all countries to estimate the Spearman’s rank correlation coefficient. Of note, the joinpoint analysis used piece-wise regression models (i.e. fitting segments of regression lines connected by joinpoints), and has been shown to provide more stable estimates than conventional regression models without joinpoints [30].

To account for latency periods between exposure and cancer diagnosis, we examined time lags ranging from 0 (no lag) to 15 years. To examine exposure during adolescence, and given the data availability, we tested the correlation of changes in cancer incidence rate among 25–34-year-olds during 2000–2012 with changes in overweight and obesity prevalence among i) 10–19-year-olds during 1985–1997 (i.e. shifting both age and calendar period by 15 years to examine exposure 15 years before cancer diagnosis (Figure S3 in the **Online Supplementary Document**), ii) 15–24-year-olds during 1990–2002 (10–year lag), iii) 20–29-year-olds during 1995–2007 (5–year lag), and iv) 25–34-year-olds during 2000–2012 (no lag), separately. Similarly, to examine exposure during adulthood, we tested the correlation of changes in cancer incidence rate among 35–49-year-olds during 2000–2012 with changes in overweight and obesity prevalence among 20–34-year-olds during 1985–1997 (15–year lag), 25–39-year-olds during 1990–2002 (10–year lag), 30–44-year-olds during 1995–2007 (5–year lag), and 35–49-year-olds during 2000–2012 (no lag). Lags longer than 15 years were not tested, because the data needed for such analysis – anthropometric measurements before 1985 and for children below 10 years of age – were sparse [11,12].

To test the validity of the above approach, we used twelve non-obesity related cancers as negative controls for the outcome variable (i.e. the nine obesity related cancers) as noted above. In addition, we conducted another negative control analysis for the exposure variable using per-capita gross national income (GNI) [32], which increased in recent decades but had no direct links to cancer incidence rate. Specifically, we replaced excess body weight with per-capita GNI, and carried out the same statistical analyses described above.

In the main analysis, we used body weight estimates from the NCD-RisC, which gathered more extensive anthropometric data than the GBD study [11,24]. Additionally, we combined both sexes due to the relatively low cancer incidence rates among young adults. Four sets of sensitivity analyses were conducted to test alternative data sources or subsamples. First, we used GBD body weight estimates (vs. NCD-RisC estimates in the main analyses). Second, for countries with occasional zero incidence (defined as less than one-third of the data being zero), we adjusted the zeros to an arbitrary small value (0.5 divided by the population size) per the joinpoint user manual [31]. This adjustment enabled the estimation of cancer annual percent changes (could not be computed for series with zeros) and allowed more countries to be included in the analysis. Third, because the incidence rates of several cancer types differed substantially between men and women, we conducted an analysis for men and women, separately, to verify the sex-combined main analysis. This was done for cancers with a >20% incidence rate difference by sex; these were cancers of the thyroid, rectum, stomach, pancreas, brain and other central nervous system, kidney, oral cavity and pharynx, bladder, and liver, melanoma of the skin, leukaemia, Hodgkin lymphoma, and non-Hodgkin lymphoma. Fourth, to examine the consistency of results in countries with higher and lower income, we ranked the 42 countries by per-capita GNI in 2012 (data from the World Bank [32]), and conducted separate analyses for the upper and lower half of the countries. While the main and sensitivity analyses used overweight and obesity combined as an indicator of excess body weight, we further tested obesity alone as an alternative indicator in a supplementary analysis.

We conducted joinpoint analysis using the Joinpoint software (version 5.0.1) [31], and all other analyses using *R* statistical language (version 4.2.1, Vienna, Austria) [33]. All of the statistical tests were two-sided, with the significance level set at *P* < 0.05.

## RESULTS

### Trends of early-onset cancers and body weight

**Figure 2:**
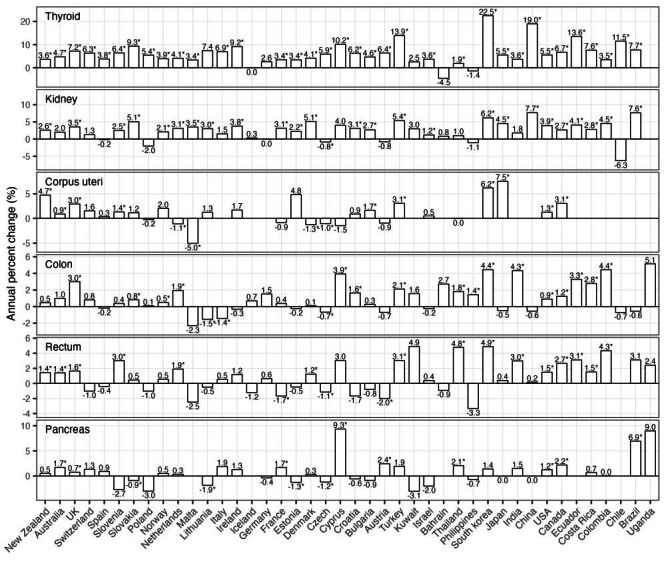
Estimated annual percent change in incidence for obesity-related cancers (age 25–49, year 2000–2012). Estimated annual percent changes are shown by both bars and numbers (asterisks indicate statistical significance, i.e. *P* < 0.05). For countries that had insufficient cancer cases for the estimation, no estimates of annual percent change are shown. This figure shows obesity-related cancers with increased incidence in more than half of the studied countries, the other obesity-related cancers are shown in Figure S4 in the **Online Supplementary Document**.

**Figure 3:**
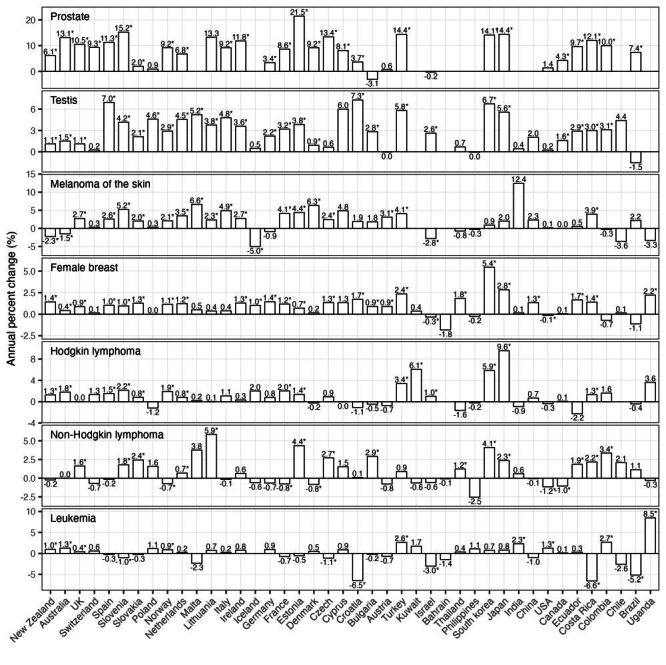
Estimated annual percent change in incidence for non-obesity-related cancers (age 25–49, year 2000–2012). Estimated annual percent changes are shown by both bars and numbers (asterisks indicate statistical significance, i.e. *P* < 0.05). For countries that had insufficient cancer cases for the estimation, no estimates of annual percent change are shown. This figure shows non-obesity-related cancers with increased incidence in more than half of the studied countries, the other non-obesity-related cancers are shown in Figure S4 in the **Online Supplementary Document**.

Of the nine cancers classified as obesity-related [10], six had increased incidence among 25–44-year-olds in more than half of the 42 countries in the CI5plus cancer database during 2000–2012 (**Figure 2**). These included cancers of the thyroid (median annual percent change across countries: 5.5%), kidney (2.7%), corpus uteri (1.2%), colon (0.7%), rectum (0.6%), and pancreas (0.5%). During the same time period, seven of the 12 non-obesity-related cancers also had increased incidence (**Figure 3**): prostate cancer (median annual percent change across countries: 9.2%), testicular cancer (2.9%), melanoma of the skin (2.0%), female breast cancer (0.9%), Hodgkin lymphoma (0.8%), non-Hodgkin lymphoma (0.6%), and leukaemia (0.4%). The remaining cancers, three obesity-related (liver, ovarian, and stomach cancers (Figure S4, Panel A in the **Online Supplementary Document**) and five non-obesity-related (cancers of the brain, oral cavity and pharynx, bladder, cervix uteri, and lung; Figure S4, Panel B in the **Online Supplementary Document**), generally did not have increases in incidence. Estimated prevalence of overweight and obesity among both adolescents (10–19-year-olds) and adults (20–49-year-olds) increased in most countries during 1985–2012 (Figure S5 in the **Online Supplementary Document**).

### Countries most affected by early-onset cancers

**Figure 4:**
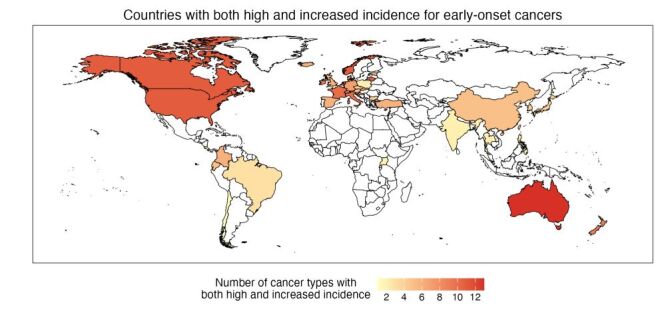
Countries most affected by early-onset cancers. To identify the most affected countries, we counted, for each country, the number of cancer types with incidence rates that were high (defined as above the median incidence among all study countries) and have increased (defined as annual percent change greater than zero). This analysis used incidence data for all 21 cancer types. Countries with no cancer incidence data are indicated by white colour.

**Figure 4** shows countries more substantially affected by early-onset cancers, identified on the basis of the number of cancer types with incidence rate above the cancer-specific median (among all study countries) and the annual percent change (>0) during the study period. These were predominantly Western countries. For example, Australia, the USA, Canada, Norway, the Netherlands, and Lithuania had more than 10 cancer types with both high (i.e. above the median) and increased incidence.

### Estimated risk association with overweight and obesity

**Figure 5:**
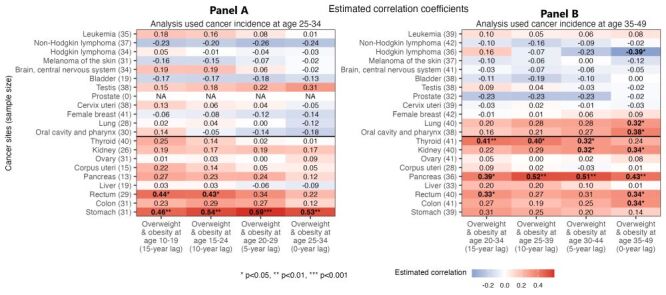
Estimated correlation between changes in body weight and changes in early-onset cancer incidence for 21 cancers. Among nine cancers classified as obesity-related (rows below the horizontal black line), six – stomach, colon, rectal, pancreatic, kidney, and thyroid cancers - had changes in cancer incidence that were positively correlated with changes in body weight (darker red cells). Numbers in the cells show Spearman’s rank correlation coefficients; bolded fonts and asterisks indicate statistical significance. For 12 cancers classified as non-obesity-related (rows above the horizontal black line), similar correlations were generally not found. These patterns hold for early-onset cancers diagnosed at age 25–34 (**Panel A**) and age 35–49 (**Panel B**), and for changes in body weight with varying time-lags (four columns for four different time-lags examined).

Among the nine obesity-related cancers, six – stomach, colon, rectal, pancreatic, kidney, and thyroid cancers – had annual percent changes among young adults positively correlated with the changes in overweight and obesity prevalence (**Figure 5**). These positive correlations were observed for overweight and obesity at different ages (including adolescence (ages 10–19) and early adulthood (ages 20–29) (**Figure 5**); and for different time lags between exposure and cancer diagnosis. For example, for overweight and obesity at age 20–34, and for a 15–year lag (i.e. cancer diagnosis at age 35–49), the estimated correlation coefficient (ρ) was 0.31 (95% CI = −0.01, 0.57; *P* = 0.056) for stomach cancer, 0.27 (95% CI = −0.04, 0.53; *P* = 0.090) for colon cancer, 0.33 (95% CI = 0.02, 0.58; *P* = 0.036) for rectal cancer, 0.39 (95% CI = 0.08, 0.64; *P* = 0.018) for pancreatic cancer, 0.22 (95% CI = −0.10, 0.50; *P* = 0.173) for kidney cancer, and 0.41 (95% CI = 0.12, 0.64; *P* = 0.007) for thyroid cancer (**Figure 5**, Table S2 in the **Online Supplementary Document**). Overall, the estimated correlations more frequently reached statistical significance for the older age group (age 35–49 vs. 25–34), potentially due to the larger numbers of countries available for analysis (**Figure 5**). For the other three cancers (liver, corpus uterine, and ovarian cancers), only weak or no correlations were found (median ρ: 0.03, 0.05, and 0.03, respectively (**Figure 5**, Figure S6 in the **Online Supplementary Document**).

For comparison, we also included twelve non-obesity-related cancers as negative controls (i.e. there should be no correlations with overweight and obesity prevalence). As noted above, seven of these cancers also had increased incidence during the study period in most studied countries (**Figure 3**). However, despite the increased incidence, for these cancers, most correlation coefficients were near zero and nonsignificant (median ρ ranged from −0.23 for prostate cancer to 0.15 for cancer of the oral cavity and pharynx (**Figure 5**, Figure S6 in the **Online Supplementary Document**). In the analysis using per-capita GNI as a negative control for excess body weight (i.e. the exposure variable), we also did not find consistent correlation with any cancer types (Figure S7 in the **Online Supplementary Document**), even though like excess body weight, per-capita GNI increased in most of the countries [32].

Similar to the main analysis, the sensitivity analyses (testing alternative data sources and subsamples) and the supplementary analysis (testing obesity alone) all found consistent positive correlations for stomach, colon, rectal, pancreatic, and kidney cancers; these analyses generally did not find such correlations for the twelve non-obesity-related cancers (Figure S6 in the **Online Supplementary Document**). For thyroid cancer, results from the sensitivity and supplementary analyses were less consistent (Figure S6 in the Online Supplementary Document).

Together, the trend analysis and correlation analysis show that four early-onset cancers (colon, rectal, pancreatic, and kidney cancers) had both increased incidence (**Figure 2**) and consistently positive correlations with excess body weight in all analyses (**Figure 5**, Figure S6 in the **Online Supplementary Document**). No clear correlations were found in the negative control analyses, supporting the specificity of the risk association with excess body weight identified for these early-onset cancers.

## DISCUSSION

We have examined incidence trends for 21 most prevalent cancers among young adults across all 42 countries included in the largest global cancer database, and the associations with the obesity epidemic. Overall, we found increased incidence for six obesity-related early-onset cancers and positive associations with excess body weight for four of these cancers (i.e. colon, rectal, pancreatic, and kidney), supporting a link between the obesity epidemic and the rising incidence in these early-onset cancers worldwide. We also found increased incidence for seven non-obesity-related early-onset cancers but no such associations with excess body weight, suggesting factors other than excess body weight were the key drivers of these other forms of early-onset malignancy. Furthermore, we found that early-onset cancers, regardless of its association with obesity, have particularly affected several Western countries (**Figure 4**), indicating that further research on early-onset cancers is most needed in these countries.

As noted in the Introduction, recent studies have reported marked increases in incidence of multiple cancer types among young adults globally [1–8]. Some researchers argued that such increases were due to increased screening, rather than a true risk increase [34]. Here, we have restricted all analyses to adults under 50, for whom population-level screening was not implemented during the study period (except for increased screening for thyroid cancer in affluent countries [35], breast cancer screening starting at age 40 in the USA [36], and a national screening programme in South Korea for thyroid, breast (targeting those ≥40 years), cervical (≥30), gastric (≥40), and hepatic cancers (≥40)) [37–39]. Thus, the incidence increases in the 13 early-onset cancers (six classified as obesity-related and seven non-obesity related (**Figure 2**, **Figure 3**) shown here were largely non-screening-related. Consistently, using US-specific data, we have found increases in the same six obesity-related cancers among young adults using a cohort-specific approach [40]. In another US-specific study, we found highly concordant temporal increases in those early-onset cancers (particularly, colon, rectum, and kidney) that were not due to increased screening [8]. Together, our studies, along with others [9,17], support true increases in early-onset cancer risk that call for an improved understanding of the underlying causes to inform early prevention.

The analyses here focus on examining the risk association with excess body weight. Although epidemiological studies have associated excess body weight with colon, rectal, pancreatic, and kidney cancers in older adults [10], the corresponding association for early-onset cancers is less clear [17]. For early-onset pancreatic cancer, a cohort study in Norway found a positive association in young men but not women [41], and for early-onset kidney cancer, a cohort study in Israel and a cohort study in Korea found a positive association [42,43]. however, specific studies remain sparse for both cancers [17]. For early-onset colorectal cancer, while a positive association with excess body weight were found in three cohort studies [18,19,44], another cohort study found no association [45]. Furthermore, the evidence supporting an impact of early-life (age 20 or earlier) excess body weight remain sparse [18–20,42]. Our study adds to the existing evidence by supporting a risk association for excess body weight starting from age 10, and extends such evidence to diverse populations across 42 countries. In addition, by investigating the association on the population level, our study provides population-level evidence that the global obesity epidemic likely has contributed to the rising incidence in select early-onset cancers (in particular, colon, rectal, pancreatic, and kidney) worldwide.

We did not find consistent associations of excess body weight with cancers of the liver, ovary, and thyroid among young adults, although these cancers were considered obesity-related in older adults [10]. It is possible that factors other than the obesity epidemic have exerted a greater influence on the trends of these cancers. Such factors may include decreased prevalence of hepatitis B virus infection (a risk factor of liver cancer) [46], increased use of oral contraceptive drugs (a protective factor of ovarian cancer) [47], and increased screening for thyroid cancer in affluent countries [35]. We also did not find consistent associations for corpus uterine cancer, which was considered obesity-related [10]. Notably, while corpus uterine cancer incidence increased the fastest in Japan (**Figure 2**), the obesity prevalence in Japan was almost unchanged in recent decades [48]. This suggests that factors other than obesity may have driven the increase, although such factors remain unknown [48].

We note several limitations of this study. First, due to the ecologic design, our analysis has limited value in inferring individual-level associations. Second, although cancer screening likely did not have a substantial impact on the young adults in our study as discussed above, potential improvements in diagnostic techniques over time could to some extent increase the diagnosed cancer cases (e.g. due to earlier detection). We were unable to account for such increases due to a lack of data. Third, although exposures very early in life (e.g. childhood) may also affect the risk of early-onset cancers [17,49], we did not test the impact of body weight before age 10, because the anthropometric data for such young ages were sparse, making the body weight estimates for this age group less reliable than those for older ages. Fourth, other limitations of the anthropometric data included that such data were scarce in less developed countries and during earlier years [11,12]. Consequently, the overweight and obesity prevalence for these populations was estimated based on certain model assumptions [11,12]. Nonetheless, our analyses used estimates from both the NCD-RisC and the GBD study, which relied on different models, and the findings were generally consistent and robust. Fifth, the CI5plus database underrepresented regions like Africa. Among the 42 countries included in the CI5plus database, 29 were in Europe and the Americas, and only one was in Africa (Table S1, Figure S1 in the **Online Supplementary Document**). Sixth, although we found an association between stomach cancer and excess body weight, we caution the interpretation of this finding because our analysis combined stomach cancers at all anatomical sites, but only cardia stomach cancer has been associated with excess body weight in previous research [10].

This study also has several strengths. As noted above, we have identified the risk association based on whether the magnitude of changes in obesity prevalence scales with the magnitude of changes in cancer incidence in a dose-response fashion, to limit spurious correlations. We have also conducted comprehensive negative control analyses, including using the twelve non-obesity-related cancers as negative controls for the outcome variable and per-capita GNI as a negative control for the exposure variable; the null results from both sets of negative control studies demonstrate the validity of our approach. Further, we have conducted multiple sensitivity and supplemental analyses, and the consistent results demonstrate the robustness of our findings. Lastly, we have tested exposures at different ages, including adolescence, and shown the risk association with excess body weight early in life including the adolescence years.

## CONCLUSIONS

Analysing data including 42 countries, we have found positive associations between the increase in excess body weight and the increase in incidence for four key early-onset cancers (i.e. colon, rectal, pancreatic, and kidney cancers). This finding suggests that the global obesity epidemic may have, in part, contributed to recent global incidence increases in these four early-onset cancers. Our finding, however, does not support associations between obesity and the other nine early-onset cancers that also increased. This calls for further research (e.g. longitudinal cohort studies) on the changing environmental exposures, childhood health conditions, and dietary patterns, which may contribute to the increases in those early-onset cancers.

## Additional material


Online Supplementary Document
