# Supplementary material for: Global obesity epidemic and rising incidence of early-onset cancers
Source: J Glob Health. 2024 Oct 11;14:04205. doi: 10.7189/jogh.14.04205 (PMC11467775; doi:10.7189/jogh.14.04205)
Supplement: Online Supplementary Document [file jogh-14-04205-s001.pdf]

Supplementary materials

Table of Contents

Table S1 2
Table S2 4
Figure S1..6
Figure S2..8
Figure S3..9
Figure S4..10
Figure S5..11
Figure S6..12
Figure S7..13

Table S1. Data availability in the 42 countries in the Cancer Incidence in Five Continents (CI5plus) database.

Continent	Country[a]	Years for analysis[b]
Oceania (2 countries)	Australia (7 registries)	1993-2012
	New Zealand	1983-2012
Americas (7 countries)	Brazil (1 registry)	1993-2012
	Canada	1983-2012
	Chile (1 registry)	1998-2012
	Colombia (1 registry)	1983-2012
	Costa Rica	1983-2011
	Ecuador (1 registry)	1985-2012
	United States[c]	1998-2012
Europe (22 countries)	Austria	1998-2012
	Bulgaria	1998-2012
	Croatia	1988-2012
	Cyprus	1998-2012
	Czech	1988-2012
	Denmark	1983-2012
	Estonia	1983-2012
	France (9 registries)	1998-2011
	Germany (2 registries)	1998-2012
	Iceland	1983-2012
	Ireland	1994-2012
	Italy (8 registries)	1998-2010
	Lithuania	1993-2012
	Malta	1993-2012
	Netherlands	1989-2012
	Norway	1983-2012
	Poland (1 registry)	1998-2012
	Slovakia	1983-2010
	Slovenia	1983-2012
	Spain (9 registries)	1993-2010
	Switzerland (6 registries)	1998-2012
	United Kingdom (11 registries)	1995-2012
Africa (1 country)	Uganda (1 registry)	1993-2012
West Asia (4 countries)	Bahrain (1 registry)	1998-2012
	Israel	1988-2012
	Kuwait (1 registry)	1998-2012
	Turkey (2 registries)	1998-2012
Southeast Asia (6 countries)	China (4 registries)	1998-2012
	India (1 registry)	1983-2012
	Japan (4 registries)	1998-2010
	South Korea (5 registries)	1999-2012
	Philippines (1 registry)	1983-2012
	Thailand (4 registries)	1993-2012

[a] For countries where the cancer registries did not cover the entire population, the number of subnational registries are listed.
[b] To ensure cancer incidence data were comparable over time, for countries with multiple subnational registries, we only included the years in which cancer incidence data were available in all registries.
[c] For the United States, data from the National Program of Cancer Registries were used.

Table S2. Estimated correlation coefficients (ρ) between changes in the prevalence of overweight and obesity combined and changes in cancer incidence.

		Analysis used cancer incidence at age 25-34							
		Overweight and obesity at age 10-19 (15-year lag)		Overweight and obesity at age 15-24 (10-year lag)		Overweight and obesity at age 20-29 (5-year lag)		Overweight and obesity at age 25-34 (0-year lag)	
**Cancer sites**	**Sample size**	**ρ (95 % CI)**	**P value**	**ρ (95 % CI)**	**P value**	**ρ (95 % CI)**	**P value**	**ρ (95 % CI)**	**P value**
Stomach	31	0.46 (0.13, 0.70)	0.009	0.54 (0.22, 0.75)	0.002	0.59 (0.30, 0.78)	0.001	0.53 (0.21, 0.74)	0.003
Colon	31	0.23 (-0.14, 0.54)	0.213	0.29 (-0.07, 0.58)	0.113	0.27 (-0.10, 0.57)	0.147	0.12 (-0.24, 0.46)	0.507
Rectum	29	0.44 (0.09, 0.69)	0.018	0.43 (0.07, 0.69)	0.021	0.34 (-0.02, 0.63)	0.068	0.22 (-0.16, 0.54)	0.261
Liver	19	0.03 (-0.43, 0.47)	0.917	0.03 (-0.43, 0.47)	0.917	-0.06 (-0.50, 0.41)	0.809	-0.09 (-0.52, 0.38)	0.726
Pancreas	13	0.27 (-0.33, 0.71)	0.373	0.23 (-0.37, 0.69)	0.448	0.24 (-0.36, 0.70)	0.426	0.12 (-0.47, 0.63)	0.710
Corpus uteri	15	0.22 (-0.32, 0.66)	0.419	0.14 (-0.40, 0.61)	0.630	0.05 (-0.48, 0.55)	0.873	0.05 (-0.47, 0.55)	0.853
Ovary	31	0.01 (-0.34, 0.37)	0.937	0.03 (-0.33, 0.38)	0.870	0.00 (-0.35, 0.36)	0.992	0.09 (-0.27, 0.43)	0.612
Kidney	26	0.19 (-0.21, 0.54)	0.346	0.17 (-0.24, 0.52)	0.413	0.19 (-0.21, 0.54)	0.352	0.17 (-0.23, 0.53)	0.395
Thyroid	40	0.25 (-0.07, 0.52)	0.126	0.14 (-0.18, 0.43)	0.390	0.02 (-0.29, 0.33)	0.888	0.01 (-0.30, 0.32)	0.948
Oral cavity and pharynx	30	0.14 (-0.24, 0.47)	0.469	-0.05 (-0.41, 0.31)	0.778	-0.14 (-0.47, 0.23)	0.466	-0.18 (-0.51, 0.19)	0.333
Lung	28	0.02 (-0.36, 0.39)	0.937	0.04 (-0.34, 0.41)	0.833	0.00 (-0.37, 0.37)	0.992	-0.12 (-0.47, 0.26)	0.536
Female breast	41	-0.06 (-0.36, 0.25)	0.695	-0.08 (-0.37, 0.24)	0.637	-0.12 (-0.41, 0.20)	0.471	-0.14 (-0.43, 0.17)	0.369
Cervix uteri	38	0.13 (-0.20, 0.43)	0.426	0.06 (-0.27, 0.37)	0.725	0.04 (-0.29, 0.35)	0.822	-0.05 (-0.37, 0.27)	0.757
Prostate	0	NA	NA	NA	NA	NA	NA	NA	NA
Testis	38	0.15 (-0.18, 0.45)	0.364	0.18 (-0.15, 0.47)	0.270	0.22 (-0.10, 0.51)	0.175	0.31 (-0.01, 0.58)	0.056
Bladder	19	-0.17 (-0.58, 0.31)	0.494	-0.17 (-0.58, 0.30)	0.475	-0.18 (-0.59, 0.30)	0.462	-0.13 (-0.55, 0.34)	0.585
Brain, central nervous system	34	0.19 (-0.16, 0.50)	0.278	0.19 (-0.16, 0.50)	0.273	0.06 (-0.28, 0.39)	0.734	-0.02 (-0.35, 0.32)	0.924
Melanoma of the skin	31	-0.16 (-0.48, 0.21)	0.400	-0.15 (-0.48, 0.21)	0.410	-0.07 (-0.42, 0.29)	0.695	-0.02 (-0.37, 0.34)	0.934
Hodgkin lymphoma	34	0.05 (-0.29, 0.38)	0.769	-0.01 (-0.34, 0.33)	0.974	-0.04 (-0.37, 0.30)	0.829	-0.03 (-0.36, 0.31)	0.871
Non-Hodgkin lymphoma	37	-0.23 (-0.51, 0.11)	0.177	-0.20 (-0.49, 0.13)	0.228	-0.26 (-0.54, 0.07)	0.121	-0.24 (-0.52, 0.09)	0.153
Leukemia	35	0.18 (-0.16, 0.49)	0.289	0.16 (-0.19, 0.47)	0.368	0.08 (-0.26, 0.40)	0.661	0.01 (-0.33, 0.34)	0.967

CI = confidence interval

Table S2 (continued).

		Analysis used cancer incidence at age 35-49							
		Overweight and obesity at age 20-34 (15-year lag)		Overweight and obesity at age 25-39 (10-year lag)		Overweight and obesity at age 30-44 (5-year lag)		Overweight and obesity at age 35-49 (0-year lag)	
**Cancer sites**	**Sample size**	**ρ (95 % CI)**	**P value**	**ρ (95 % CI)**	**P value**	**ρ (95 % CI)**	**P value**	**ρ (95 % CI)**	**P value**
Stomach	39	0.31 (-0.01, 0.57)	0.056	0.25 (-0.07, 0.53)	0.121	0.20 (-0.12, 0.49)	0.216	0.14 (-0.18, 0.44)	0.381
Colon	41	0.27 (-0.04, 0.53)	0.090	0.19 (-0.12, 0.47)	0.230	0.25 (-0.07, 0.51)	0.121	0.34 (0.03, 0.58)	0.032
Rectum	40	0.33 (0.02, 0.58)	0.036	0.27 (-0.04, 0.54)	0.089	0.31 (0.00, 0.57)	0.053	0.34 (0.03, 0.59)	0.032
Liver	33	0.20 (-0.15, 0.51)	0.256	0.20 (-0.15, 0.51)	0.260	0.10 (-0.25, 0.43)	0.585	0.01 (-0.33, 0.35)	0.950
Pancreas	36	0.39 (0.08, 0.64)	0.018	0.52 (0.23, 0.72)	0.001	0.51 (0.22, 0.72)	0.002	0.43 (0.12, 0.66)	0.009
Corpus uteri	28	0.09 (-0.29, 0.45)	0.640	0.02 (-0.36, 0.39)	0.935	-0.03 (-0.40, 0.35)	0.895	0.01 (-0.37, 0.38)	0.972
Ovary	41	0.05 (-0.26, 0.36)	0.740	0.00 (-0.30, 0.31)	0.980	0.02 (-0.29, 0.33)	0.890	0.08 (-0.23, 0.38)	0.597
Kidney	40	0.22 (-0.10, 0.50)	0.173	0.29 (-0.02, 0.56)	0.065	0.32 (0.01, 0.57)	0.046	0.34 (0.03, 0.59)	0.034
Thyroid	41	0.41 (0.12, 0.64)	0.007	0.40 (0.10, 0.63)	0.010	0.32 (0.01, 0.57)	0.043	0.24 (-0.07, 0.51)	0.125
Oral cavity and pharynx	38	0.16 (-0.17, 0.45)	0.347	0.21 (-0.12, 0.49)	0.213	0.27 (-0.06, 0.54)	0.102	0.38 (0.07, 0.63)	0.018
Lung	40	0.20 (-0.12, 0.48)	0.221	0.28 (-0.04, 0.54)	0.085	0.28 (-0.03, 0.54)	0.079	0.32 (0.00, 0.57)	0.048
Female breast	42	-0.01 (-0.31, 0.29)	0.949	0.01 (-0.29, 0.31)	0.940	0.06 (-0.25, 0.36)	0.709	0.09 (-0.22, 0.38)	0.570
Cervix uteri	39	-0.03 (-0.34, 0.29)	0.848	0.02 (-0.30, 0.33)	0.917	-0.01 (-0.33, 0.30)	0.943	-0.03 (-0.34, 0.29)	0.863
Prostate	32	-0.23 (-0.53, 0.13)	0.215	-0.23 (-0.54, 0.12)	0.196	-0.23 (-0.54, 0.13)	0.204	-0.02 (-0.37, 0.33)	0.913
Testis	38	0.09 (-0.23, 0.40)	0.575	0.04 (-0.29, 0.35)	0.829	-0.03 (-0.35, 0.29)	0.840	-0.02 (-0.34, 0.30)	0.908
Bladder	38	-0.11 (-0.42, 0.21)	0.495	-0.19 (-0.48, 0.14)	0.262	-0.10 (-0.40, 0.23)	0.560	0.00 (-0.32, 0.32)	0.987
Brain, central nervous system	41	-0.03 (-0.33, 0.28)	0.861	-0.07 (-0.37, 0.24)	0.641	-0.06 (-0.36, 0.26)	0.731	-0.05 (-0.35, 0.26)	0.763
Melanoma of the skin	37	-0.10 (-0.41, 0.24)	0.570	-0.06 (-0.38, 0.27)	0.710	0.00 (-0.32, 0.33)	0.988	-0.12 (-0.43, 0.21)	0.485
Hodgkin lymphoma	36	0.16 (-0.18, 0.47)	0.342	-0.07 (-0.39, 0.27)	0.701	-0.23 (-0.52, 0.11)	0.185	-0.39 (-0.64, -0.07)	0.018
Non-Hodgkin lymphoma	42	-0.10 (-0.39, 0.21)	0.521	-0.16 (-0.45, 0.15)	0.299	-0.09 (-0.39, 0.22)	0.561	-0.02 (-0.32, 0.29)	0.908
Leukemia	39	0.10 (-0.22, 0.40)	0.547	0.05 (-0.27, 0.36)	0.775	0.06 (-0.26, 0.37)	0.725	0.08 (-0.24, 0.39)	0.610

CI = confidence interval

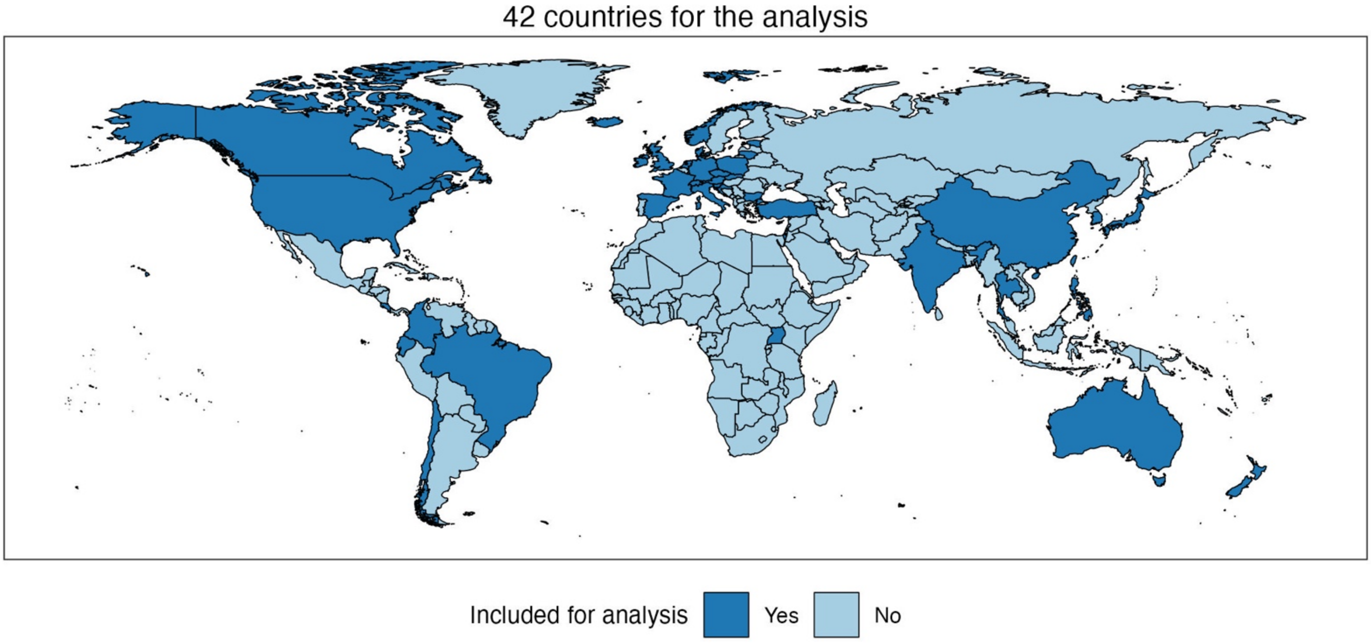

Figure S1. Countries included in the Cancer Incidence in Five Continents data and this analysis.

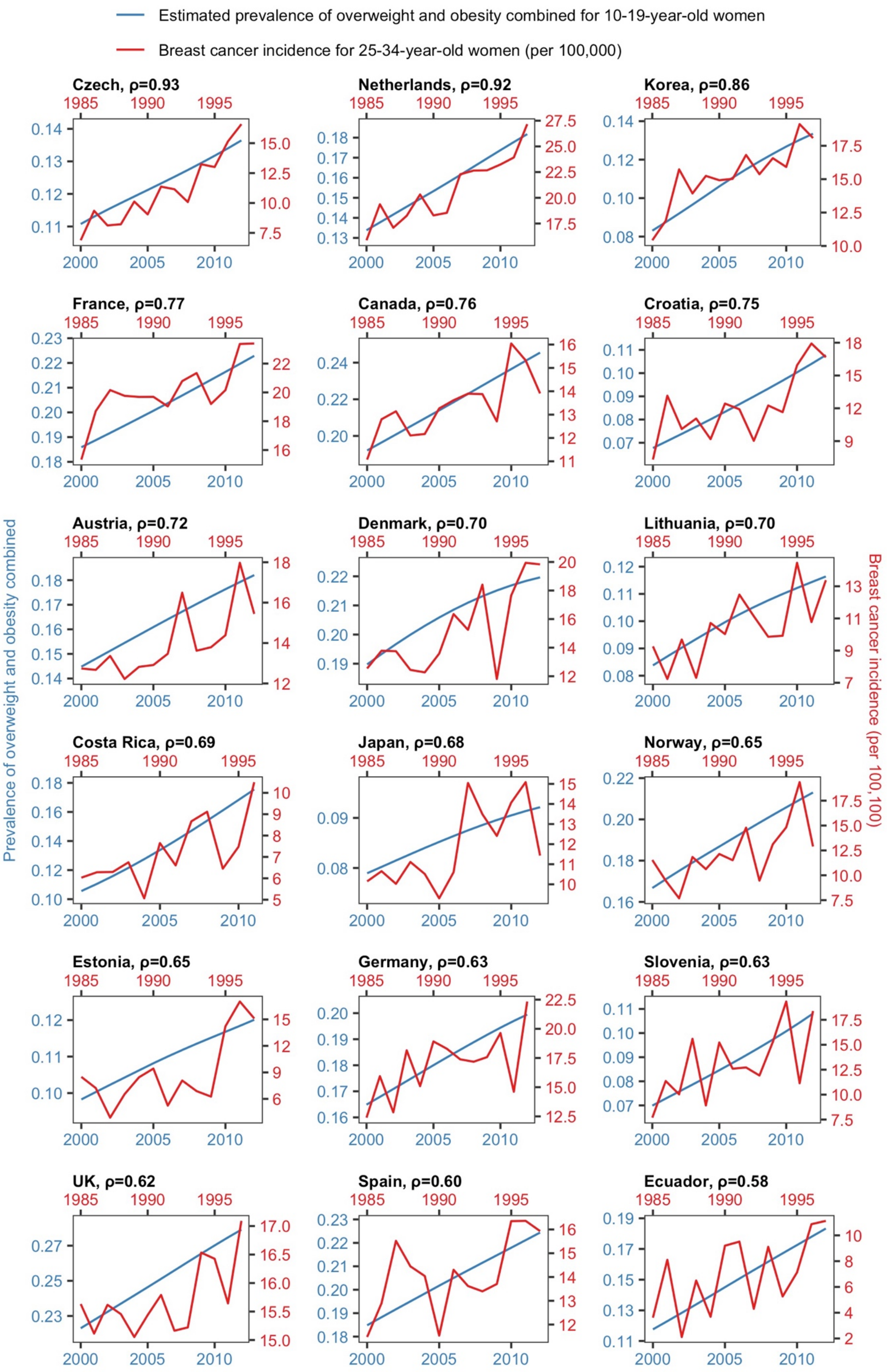
Estimated prevalence of overweight and obesity combined for 10-19-year-old women
Breast cancer incidence for 25-34-year-old women (per 100,000)
Czech, ρ=0.93
Netherlands, ρ=0.92
Korea, ρ=0.86
France, ρ=0.77
Canada, ρ=0.76
Croatia, ρ=0.75
Austria, ρ=0.72
Denmark, ρ=0.70
Lithuania, ρ=0.70
Costa Rica, ρ=0.69
Japan, ρ=0.68
Norway, ρ=0.65
Estonia, ρ=0.65
Germany, ρ=0.63
Slovenia, ρ=0.63
UK, ρ=0.62
Spain, ρ=0.60
Ecuador, ρ=0.58
Prevalence of overweight and obesity combined
Breast cancer incidence (per 100,100)

Figure S2. Estimated correlation between the prevalence trend for overweight and obesity combined and the incidence trend for breast cancer. The analysis here considered a 15-year lag between exposure to overweight and obesity (blue lines) and breast cancer incidence (red lines). The estimated Spearman's rank correlation coefficient (ρ) for each country is shown on the top of each panel. This analysis found strong positive correlation between excess bodyweight and breast cancer in many countries (the 18 countries with the strongest estimated correlations are shown), even though previous studies have shown a negative association in premenopausal women (see Ref10 of the main text).

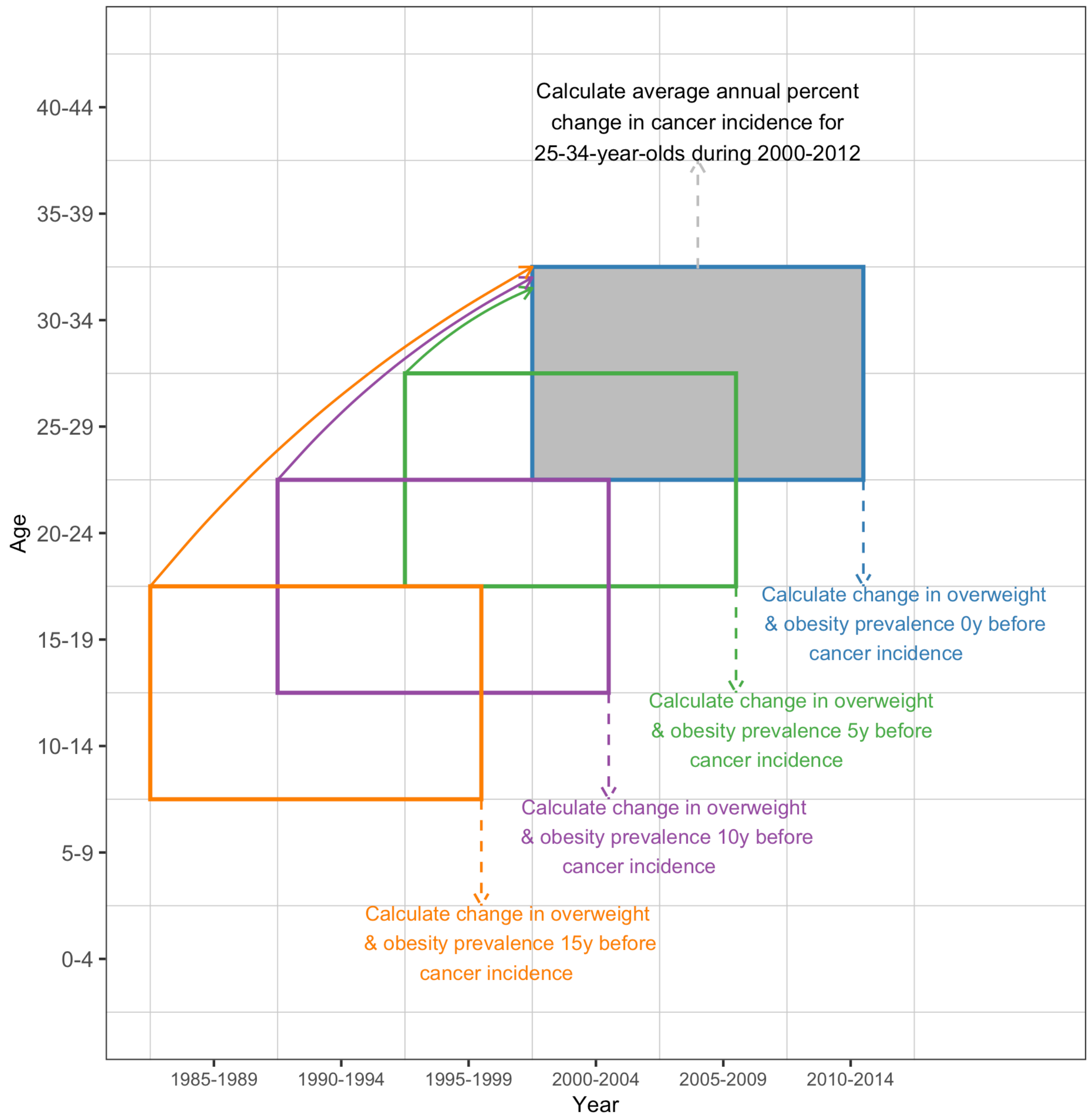

Figure S3. Age groups and calendar periods for analysis. To test the impact of exposure during adolescence, and to allow for a 15-year lag between the exposure and cancer incidence, we tested whether changes in overweight and obesity prevalence among 10-19-year-olds during 1985-1997 (orange rectangle) correlated with changes in cancer incidence among 25-34-year-olds during 2000-2012 (grey area). Similarly, we also tested 10-year lag using overweight and obesity prevalence among 15-24-year-olds during 1990-2002 (purple rectangle), 5-year lag using overweight and obesity prevalence among 20-29-year-olds during 1995-2007 (green rectangle), and 0-year lag using overweight and obesity prevalence among 25-34-year-olds during 2000-2012 (blue rectangle).

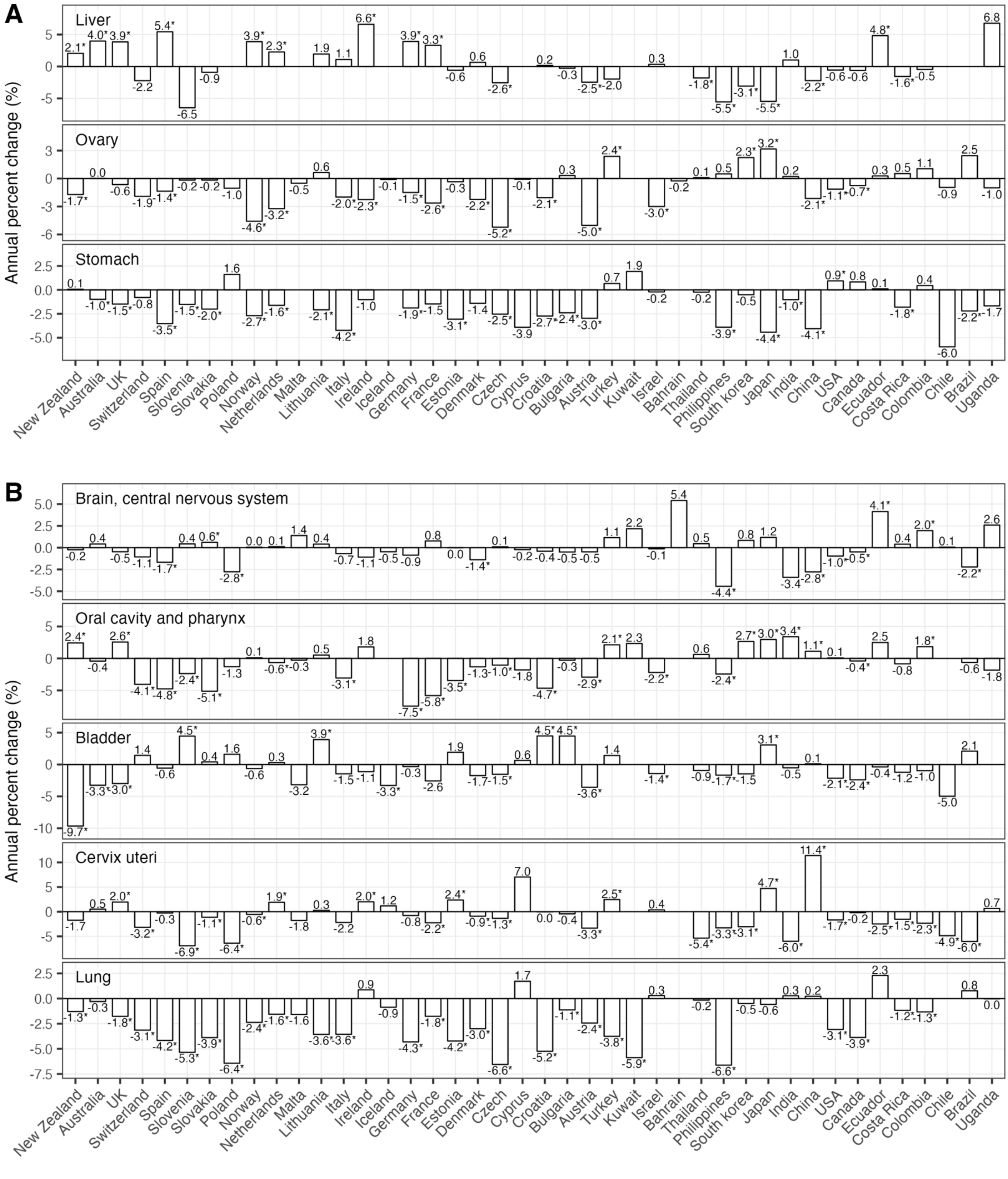

Figure S4. Same as Figures 2-3 but showing the obesity-related (panel A) and non-obesity-related cancers (panel B) that had increased incidence in no more than half of the 42 countries.

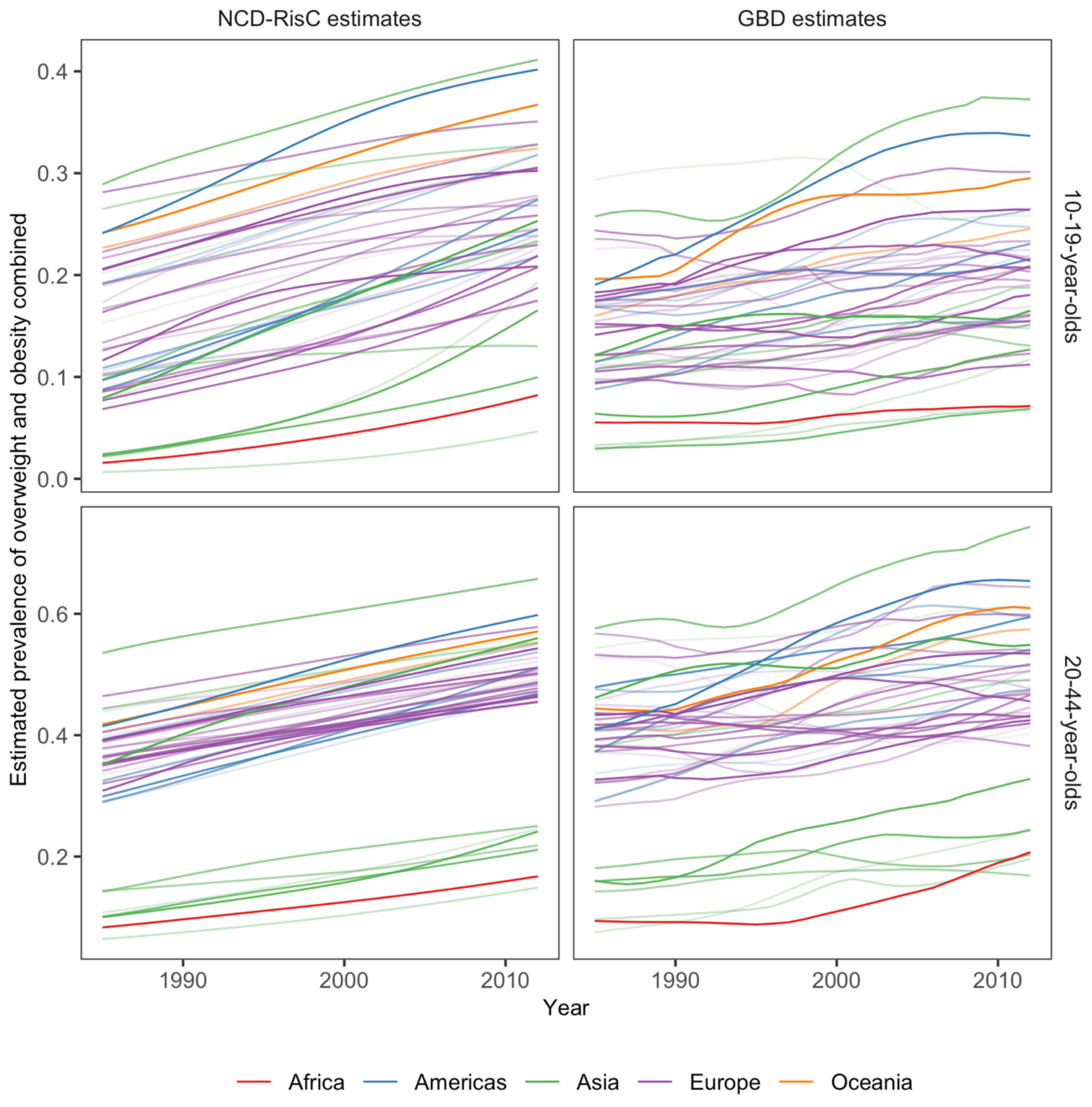

Figure S5. Estimated prevalence of overweight and obesity combined in 42 countries. Each line shows one country. Countries in different continents were differentiated by different colors, and countries on a same continent were differentiated by different shades of the same color. NCD-RisC = Non-communicable diseases risk factor collaboration; GBD = Global burden of disease.

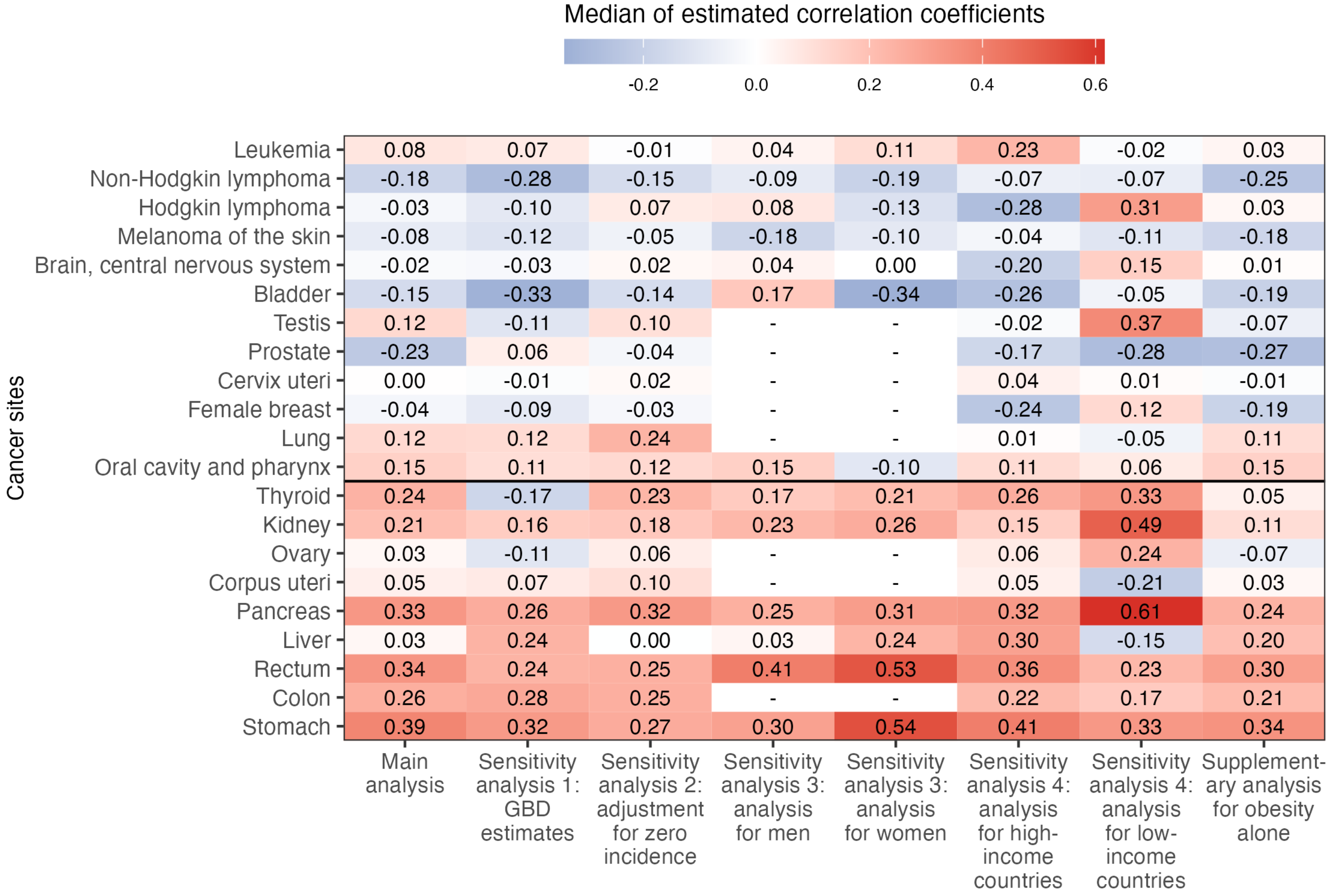

Cancer sites	Main analysis	Sensitivity analysis 1: GBD estimates	Sensitivity analysis 2: adjustment for zero incidence	Sensitivity analysis 3: analysis for men	Sensitivity analysis 3: analysis for women	Sensitivity analysis 4: analysis for high-income countries	Sensitivity analysis 4: analysis for low-income countries	Supplement-ary analysis for obesity alone
Leukemia	0.08	0.07	-0.01	0.04	0.11	0.23	-0.02	0.03
Non-Hodgkin lymphoma	-0.18	-0.28	-0.15	-0.09	-0.19	-0.07	-0.07	-0.25
Hodgkin lymphoma	-0.03	-0.10	0.07	0.08	-0.13	-0.28	0.31	0.03
Melanoma of the skin	-0.08	-0.12	-0.05	-0.18	-0.10	-0.04	-0.11	-0.18
Brain, central nervous system	-0.02	-0.03	0.02	0.04	0.00	-0.20	0.15	0.01
Bladder	-0.15	-0.33	-0.14	0.17	-0.34	-0.26	-0.05	-0.19
Testis	0.12	-0.11	0.10	-	-	-0.02	0.37	-0.07
Prostate	-0.23	0.06	-0.04	-	-	-0.17	-0.28	-0.27
Cervix uteri	0.00	-0.01	0.02	-	-	0.04	0.01	-0.01
Female breast	-0.04	-0.09	-0.03	-	-	-0.24	0.12	-0.19
Lung	0.12	0.12	0.24	-	-	0.01	-0.05	0.11
Oral cavity and pharynx	0.15	0.11	0.12	0.15	-0.10	0.11	0.06	0.15
Thyroid	0.24	-0.17	0.23	0.17	0.21	0.26	0.33	0.05
Kidney	0.21	0.16	0.18	0.23	0.26	0.15	0.49	0.11
Ovary	0.03	-0.11	0.06	-	-	0.06	0.24	-0.07
Corpus uteri	0.05	0.07	0.10	-	-	0.05	-0.21	0.03
Pancreas	0.33	0.26	0.32	0.25	0.31	0.32	0.61	0.24
Liver	0.03	0.24	0.00	0.03	0.24	0.30	-0.15	0.20
Rectum	0.34	0.24	0.25	0.41	0.53	0.36	0.23	0.30
Colon	0.26	0.28	0.25	-	-	0.22	0.17	0.21
Stomach	0.39	0.32	0.27	0.30	0.54	0.41	0.33	0.34

Figure S6. Median of estimated correlation coefficients in the main, sensitivity, and supplementary analyses. Our analysis estimated the correlation between changes in overweight and obesity prevalence and changes in cancer incidence among 42 countries. For each set of analysis (main/sensitivity/supplementary) and for each cancer, we pooled all correlation coefficients estimated for the exposure tested at different ages and different time lags between exposure and cancer incidence, and used the median as a summary statistics (shown by both colors and numbers in each cell). NCD-RisC = Non-communicable disease risk factor collaboration. GBD = Global burden of disease.

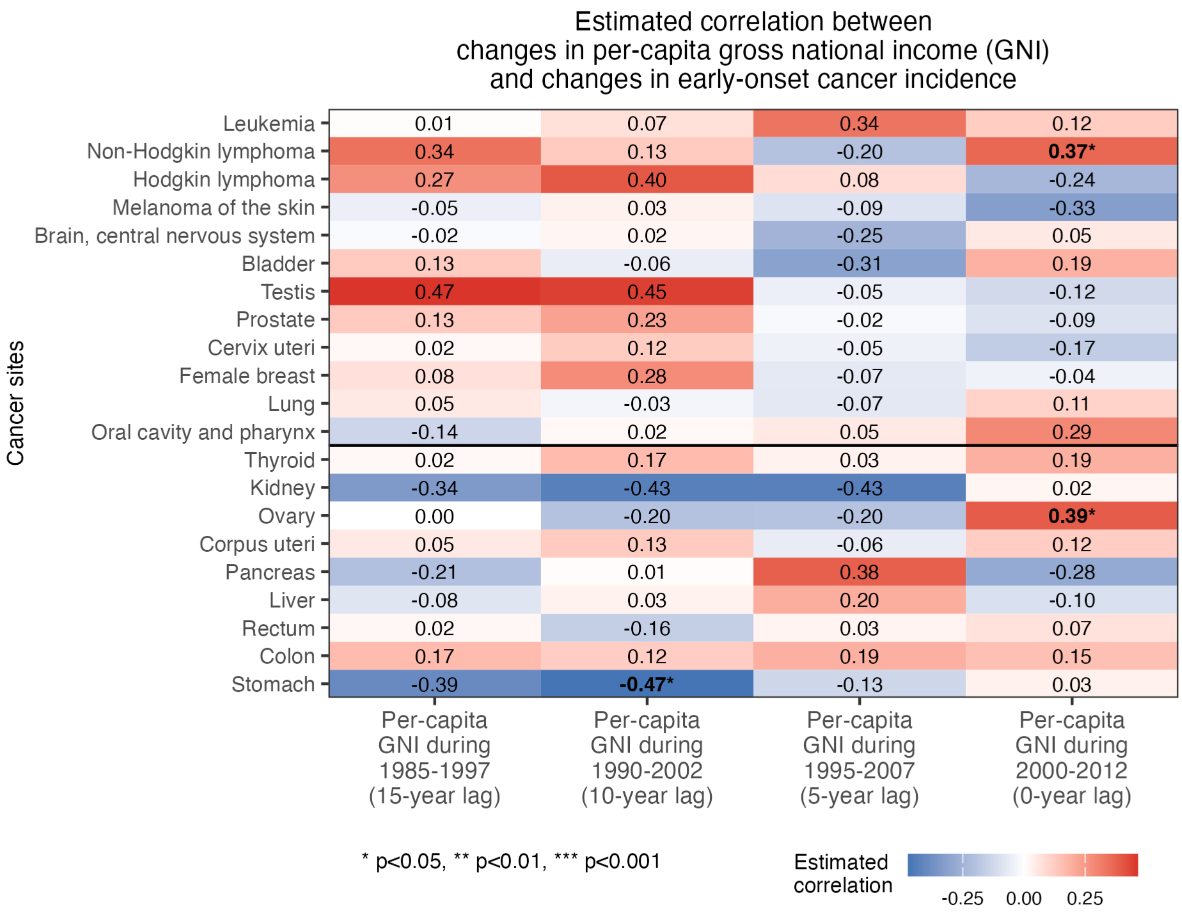

Figure S7. Estimated correlation between changes in per-capita gross national income (GNI) and changes in early-onset cancer incidence. The analysis was carried out using the same procedure as in the main analysis (see Figure 5), but replaced excess body weight (the exposure of interest) with per-capita gross national income (a variable that increased in recent decades but had no direct links to cancer incidence rates).
